# Comparative analysis of five type II TA systems identified in *Pseudomonas aeruginosa* reveals their contributions to persistence and intracellular survival

**DOI:** 10.3389/fcimb.2023.1127786

**Published:** 2023-02-13

**Authors:** Yingjie Song, Hong Tang, Rui Bao

**Affiliations:** ^1^ College of Life Science, Sichuan Normal University, Chengdu, China; ^2^ Division of Infectious Diseases, State Key Laboratory of Biotherapy and Center of Infectious Diseases, West China Hospital, Sichuan University, Chengdu, China

**Keywords:** P. aeruginosa isolates, toxin-antitoxin system, persistence, invasion ability, intracellular survival

## Abstract

**Background:**

*Pseudomonas aeruginosa* is a grave nosocomial pathogen that persistently inhabits the lungs of patients with cystic fibrosis (CF) and causes various chronic infections. The bacterial toxin–antitoxin (TA) system is associated with latent and long-term infections, but the underlying mechanisms remain to be fully characterized.

**Methods:**

We here investigated the diversity and function of five genomic type II TA systems widely distributed among *P. aeruginosa* clinical isolates. We also examined the distinct structural features of the toxin protein from different TA systems and characterized their contributions to persistence, invasion ability, and intracellular infection caused by *P. aeruginosa*.

**Results:**

ParDE, PA1030/PA1029, and HigBA could modulate persister cell formation under treatment with specific antibiotics. Furthermore, cell-based transcriptional and invasion assays revealed that PA1030/PA1029 and HigBA TA systems were critical for intracellular survival.

**Discussion:**

Our results highlight the prevalence and diverse roles of type II TA systems in *P. aeruginosa* and evaluate the possibility of using PA1030/PA1029 and HigBA TA pairs as targets for novel antibiotic treatments.

## Introduction

*Pseudomonas aeruginosa* is a major gram-negative opportunistic pathogen that can cause fatal infections in immunocompromised patients (Lyczak et al., 2000; Wu et al., 2015). It has specifically evolved to acclimatize to stressful environmental conditions and is frequently isolated from patients with burn infection and nosocomial pneumonia (Sato et al., 1988). *P. aeruginosa* infections may eventually establish and develop into serious life-threatening diseases such as ventilator-associated pneumonia and cystic fibrosis (CF) (Lyczak et al., 2000). *P. aeruginosa* has developed resistance to various antibiotics and has therefore been declared as one among the ESKAPE bacteria (together with *Enterococcus faecium*, *Staphylococcus aureus*, *Klebsiella pneumoniae*, and *Acinetobacter baumannii*), which are listed as critical priority pathogens by the World Health Organization (Qin et al., 2022). Conventional treatment of *P. aeruginosa* infection is extremely difficult because *P. aeruginosa* develops resistance to antibiotics through the formation of persister cells, a transient dormant state for surviving at above lethal antibiotic concentrations without acquiring resistance-related mutations (Mulcahy et al., 2010). Therefore, studying the mechanism of persister formation could facilitate new drug development against *P. aeruginosa* infection and reduce the emergence of multidrug-resistant strains.

While multiple pathways, including general stress response, DNA repair, energy metabolism alteration, post-transcriptional regulation, and protein level modification, have been considered to be related to the bacterial persistence mechanism, increasing evidence has suggested that the bacterial toxin–antitoxin (TA) system plays key roles in persister formation (Wang and Wood, 2011; Fasani and Savageau, 2013). A typical TA system consists of a stable toxin that causes growth arrest by interfering with vital processes and a cognate antitoxin that confers immunity to the toxin protein/RNA (Fraikin et al., 2020). Until now, according to the primary sequence of the toxins and their action mode, I–VIII TA systems have been classified (Jurėnas et al., 2022). Among them, type II TA systems are the most diverse and extensively studied. The toxin/antitoxin pair from the type II TA family are typical proteins tightly associated with the TA complex under non-stress conditions, in which the antitoxin acts as a translational repressor by binding at the upstream of TA loci (Kamruzzaman et al., 2021). Stress-induced antitoxin degradation could release the cognate toxin, which then inhibits diverse essential cellular processes, thereby leading to bacterial death for protecting the cell population or to bacterial persistence. In general, the type II TA system is a crucial strategy for enhancing stress tolerance and is recognized as a promising therapeutic target against antibiotic-resistant pathogenic bacteria (Alonso, 2021). While the emergence of novel TA members and reports regarding new type II TA functions suggest that many unknown mechanisms remain to be explored.

According to the TADB 2.0 database, five pairs of type II TA cassettes have been identified in the *P. aeruginosa* PAO1 genome: PA0124/PA0123 (denoted as ParDE, *par* operon in the RK2 plasmid), PA4674.1/PA4674 (denoted as HigBA, host inhibition of growth), PA1030/PA1029, PA1878/PA1879, and PA3270/PA3269 (Xie et al., 2018). The increase in their expression levels were associated with ciprofloxacin- and colistin-induced persister cell formation by *P. aeruginosa* isolates (Golmoradi Zadeh et al., 2022). The toxin ParE protects *P. aeruginosa* against the quinolone and other antibiotics by inhibiting gyrase-mediated DNA supercoiling, while higher ParE concentrations are also themselves toxic to cells (Muthuramalingam et al., 2019). Although both ParE and HigB belong to the RelE/ParE superfamily and adapt similar folds, HigB functions as an endonuclease-type toxin that cleaves ribosome-bound mRNAs (Wood and Wood, 2016). Evidence has revealed that HigB contributes to persister formation under ciprofloxacin treatment and upregulates the expression of type III secretion system (T3SS) genes (Li et al., 2016). The toxin PA1030 is a putative NAD^+^ phosphorylase having the classical (R-E-S) active site (Zhou et al., 2021). By contrast, although PA1878 and PA3270 have been classified into VapC and GNAT families, respectively, little is known about their catalytic sites and functions. Regarding cellular functions, through experiments, HigB alone has been shown to be involved in pyoverdine synthesis, biofilm formation, and bacterial infection (Wood and Wood, 2016).

We here explored the prevalence and biological function of the five genomic type II TA systems by using a combination of biochemical, structural biology, and microbiological methods. The results revealed that, except for HigBA, all other type II TA systems are normally conserved in 4955 P. *aeruginosa* clinical isolates, and the *higB* gene is lost in nearly a quarter of the strains. Overexpression of any single toxin alone would cause rapid growth arrest, whereas mutations in active sites could significantly reduce their toxic effects. With meropenem or cephalosporin treatment, ParDE, PA1030/PA1029, and HigBA modulated persister formation. Furthermore, cell-based transcriptional and invasion assays revealed that PA1030/PA1029 and HigBA TA systems were activated under conditions that closely mimicked the CF sputum and were critical for intracellular survival. This study characterized the different functions of the five genomic type II TAs in *P. aeruginosa*, particularly their discrete involvements or contributions to antibiotic resistance, bacterial persistence, and virulence. Understanding the details of these type II TA systems will offer new target genes that can be investigated further for developing better strategies to treat *P. aeruginosa* infection.

## Materials and methods

### Bacterial strains, medium, and growth conditions

The fragments of toxin and cognate antitoxin genes were amplified from *P. aeruginosa* PA14 genomic DNA and subcloned into the plasmid pRSFDuet-1 which has two multiple cloning sites (toxin for T7 promoter-1, antitoxin for T7 promoter-2). *E. coli* BL21 (DE3) hosts harboring pRSFDuet-based plasmids were cultured in Luria broth (LB) medium overnight, then the cells were reinoculated into fresh LB at OD_600_ = 0.1 and continued to cultivate at 37°C to different growth phases supplemented with 0.1 mM IPTG (Song et al., 2022). The cells were serially diluted and the dilutions were spotted on LB agar plates containing 0.1 mM IPTG. For MIC studies, overnight bacterial cultures of WT *P. aeruginosa* and mutants were re-inoculated into fresh M63 minimal medium supplemented with magnesium sulfate and arginine. For qRT-PCR and invasion studies, overnight bacterial cultures of WT *P. aeruginosa* and mutants were re-inoculated into fresh LB and grown to an OD_600_ of 1.0. All the bacteria strains and plasmids used in this study were detailed in **Supporting Table S1**.

### Basic local alignment search of five type II TA systems within *P. aeruginosa* isolates

The basic information of TA loci and genes in *P. aeruginosa* PAO1 were obtained from TADB 2.0 database (https://bioinfo-mml.sjtu.edu.cn/TADB2/index.php) and the detailed amino acid sequences were collected from Uniprot (https://www.uniprot.org/). The DIAMOND BLASTX search tool was used to find the number and family of similar type II TA loci, for a total of 4955 P*. aeruginosa* isolates in Pseudomonas Genome database (https://www.pseudomonas.com/blast/setdiamondblastx). The E-value cut-off was set at 1e-12 with previously computationally and experimentally identified *P. aeruginosa* proteins, the query coverage and identity cut-off were set at 30% and 70%, respectively. The ORF files and gene assemblies of outcomes were also analyzed to remove false TA pairs.

### Construction of *P. aeruginosa* deletion mutants

Briefly, the two-step allelic exchange method was used to construct *P. aeruginosa* mutant strains (Song et al., 2021b). The upstream (800 bp) and downstream (800 bp) fragments of toxin genes from the *P. aeruginosa* PA14 were subcloned into the suicide plasmid pEX18Gm. These vectors were first transformed into *E. coli* S17-1 and mobilized into *P. aeruginosa* PA14 through incubating with S17-1. The colonies were firstly screened in LB agar containing 50 µg/ml gentamicin and then in 10% sucrose agar by using sucrose-mediated counter-selection, and further identified by PCR. All the primers used in this work are listed in **Table S2**.

### MBC-B determination

We used minimal bactericidal concentration of an antimicrobial agent for biofilm cells (MBC-B), an improved method based on MIC to mimic antibiotic treatments of established biofilm infections (Mah, 2014). Overnight bacterial cultures of WT *P. aeruginosa* and mutants were re-inoculated into fresh M63 minimal medium supplemented with magnesium sulfate and arginine at OD_600_ = 0.1, then the dilution was added in a 96-well microtiter dish for 24 h at 37°C. Prepare a 10 × dilution series of antibiotic for 7 wells, the initial concentrations of meropenem, tobramycin, azithromycin, gentamicin, ciprofloxacin, polymyxin B, cephalosporin are as follows: 0.1 mg/ml, 0.5 mg/ml, 0.5 mg/ml, 0.1 mg/ml, 1 mg/ml, 0.1 mg/ml, 0.1 mg/ml. The spent supernatant in wells was removed and refilled with 10 µl each antibiotic dilutions and 90 µl fresh M63 minimal medium for 24 h at 37°C. Next, the spent supernatant was removed and added 115 µl fresh M63 minimal medium for 24 h at 37°C. The MBC-B was determined by plating on LB agar plates for 16 h at 37°C.

### Persistence assay

Persistence of *P. aeruginosa* was measured by time-dependent killing experiments as previously reported (Pinel-Marie et al., 2021). Overnight bacterial cultures of WT *P. aeruginosa* and mutants were re-inoculated into fresh LB medium at OD_600_ = 0.1 and grown to an OD_600_ of 1.0. Then the bacterial cultures were exposed to 50 µg/ml meropenem, 100 µg/ml cephalosporin, 5 µg/ml tobramycin or 5 µg/ml ciprofloxacin. Cell viability (CFU/ml) was determined by serial dilution and plating at the time points including 0, 4, 12, 24 and 36 hours. The plate was incubated at 37°C for 16 h before colony counting.

### RNA preparation and qRT-PCR assays

Overnight bacterial cultures of WT *P. aeruginosa* grown in LB medium were re-inoculated into fresh SCFM at OD_600_ = 0.5 for next culture at 37°C for 0.5 or 1 h. Then the bacteria cultures were collected and the bacterial RNA was extracted by using Trizol (Invitrogen, USA) (Rio et al., 2010). As for bacterial RNA isolation from A549 cells, the A549 cells were seeded in a 6-well tissue culture plate and then were infected with *P. aeruginosa* PA14 at an MOI of 100 for 1 and 2 h, respectively (Song et al., 2021b). The A549 cells were treated with 1% Triton X-100 for 15 min, washed with PBS twice, and then treated with DNase for another 15 min. The bacteria were collected for RNA isolation. PrimeScript™ RT reagent Kit (TaKaRa, Beijing) was used to synthesize the cDNA and 2× ChamQ™SYBR^®^qPCR Master Mix (Vazyme, Nanjing) was used to perform qPCR assays. The detailed reaction system was 2 × ChamQ SYBR qPCR Master Mix (Without ROX) 10 μl, Primer 1 (10 μM) 0.4 μl, Primer 2 (10 μM) 0.4 μl, Template cDNA 1 μg and ddH_2_O up to 20 μl. The detailed qPCR procedure used in study was: stage 1 (95°C 30 seconds, repeat once), stage 2 (95°C 10 seconds, 60°C 30 seconds, repeat for 40 times) and stage 3 (95°C 15 seconds, 60°C 60 seconds, 95°C 15 seconds, repeat once). The *oprL* gene was used as a normalizer. All the primers used in this study were detailed in **Supporting Table S3**.

### Cell adhesion and invasion assay

Briefly, The WT *P. aeruginosa* and mutants were grown to an OD_600_ at 0.6 and counted by serial dilution and plate counts (Song et al., 2021b). The A549 cells (obtained from ATCC) were seeded at 2 × 10^5^ cells per well in a 24-well tissue culture plate and then infected with 1 × 10^6^ cells of *P. aeruginosa* strains for 0.5 h, then the cells were washed three times with PBS and lysed by adding 0.5% Triton X-100 for serial dilution and plating on LB agar plates, sterile PBS as negative control. As for invasion assays, the *P. aeruginosa* strains were infected with cells at a MOI of 10 and kept for 1 h at 37°C. The cells were washed three times with PBS and incubated for another 2 h in DMEM containing gentamicin (150 μg/ml) to kill extracellular *P. aeruginosa*. Finally, the cells were washed with PBS, lysed in 0.5% Triton X-100, and then diluted with PBS for CFU counting on LB agar plates.

### Macrophage uptake and intracellular survival

Intracellular survival assay was performed as previously reported (Helaine et al., 2014; Paul et al., 2022). The RAW264.7 macrophages were seeded at 2 × 10^5^ density/well on a 24-well plate, and the *P. aeruginosa* strains were infected with cells at a MOI of 10 and cultured in DMEM for 16–18 h. The cells were washed with sterile PBS and cultured in fresh medium containing 100 μg/ml kanamycin for 2 h. Then the macrophage cells were lysed in 0.5% Triton X-100 for serial dilution and plating on LB agar plates. As for intracellular survival, the macrophage cells were lysed for serial dilution at 24 h post infection, then repeated the operation as above.

## Results

### Bioinformatics-based analysis of the genetic organization and intracellular networks of the widely distributed type II TAs in *P. aeruginosa*

The TADB 2.0 database is an up-to-date and useful resource for identifying and characterizing type II TA systems in bacteria and archaea (Xie et al., 2018). This database offers comprehensive information about the predicted and experimentally supported TA loci and allows comparative analysis of the given TA modules across relative genomes. TADB 2.0 has currently identified five pairs of type II TA systems in the chromosomes of the well-studied *P. aeruginosa* PAO1, PA7, and PA14. The localization of these systems in the complete PAO1 genome and related sequence information are presented in **Supplementary Figure 1**. Based on the comparative analysis, PA0124/PA1025, PA1878/PA1879, PA3270/PA3269, and PA4674.1/PA4674 were found to be homologous to the type II TA systems ParDE, VapBC, GNAT/Xre, and HigBA, respectively, whereas PA1030/PA1029 has not yet been characterized. To evaluate their presence across different clinical isolates, we used the DIAMOND BLASTX search tool (E-value cut-off 1e-12, 30% query coverage, and 70% identity cut-off) against the Pseudomonas Genome DataBase (Winsor et al., 2016). The results revealed that all available genomes of the 4955 P*. aeruginosa* isolates possess ParDE, PA1030/PA1029, PA1878/PA1879, and PA3270/PA3269 with high sequence identity (**Table 1**, **Table S3**), particularly the ParDE loci found in more than 4800 strains with >98% homology. The only exception is HigBA, in which the toxin gene *higB* is lost in 1731 strains, but its cognate antitoxin gene *higA* remains in all isolates and retains a high homology (4124 strains for identity ≥ 98%, and 820 strains for 70 ≤ identity < 98%). The divergent genetic evolution of *higBA* pair homologs has also been observed *in A. aceti*, *A. persici*, and *Kozakia baliensis*, in which *higA* is more reserved than *higB*. This suggests an additional regulatory role of the antitoxin (Xia et al., 2021). This result is consistent with that of our previous studies, which showed that HigA could act as a diverse repressor rather than just an antitoxin regulating extended genome regions and involving in more pathways (Song et al., 2021a).

**Table 1:** Screening genes homologous to type II TA of *P. aeruginosa* PAO1 among 4955 P*. aeruginosa* clinical isolates.

Gene	Number of homologous genes in *P. aeruginosa* isolates		
	identify ≥ 98%	70 ≤ identify < 98%	30 ≤ identify < 70%
PA0124 (*parE*)	4836	159	0
PA0125 (*parD*)	4969	24	0
PA1029	4893	112	0
PA1030	4674	314	0
PA1878	4891	76	0
PA1879	4500	482	14
PA3269	4685	275	0
PA3270	4964	30	0
PA4674.1 (*higB*)	3068	148	8
PA4674 (*higA*)	4124	820	10

For each TA pair its sequence identification compared to TA loci in P. aeruginosa PAO1 is stated in % and classified into three parts.

**Figure 1:**
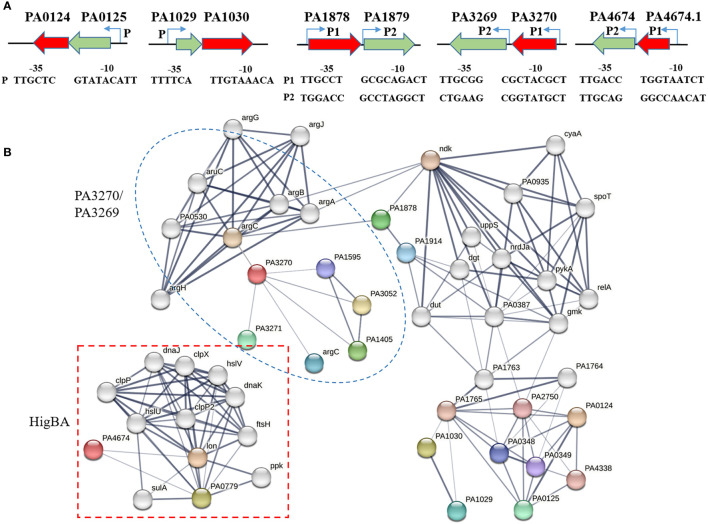
Genomic loci and link network of type II TA genes. **(A)** The operons of type II TA systems and relative promoters. The putative –35 and –10 regions of the two promoters are predicted by using Softberry online programs (http://linux1.softberry.com/) and underlined. **(B)** Network showing the interactions between toxin and other proteins, available in the STRING database. The parameter of interactive relationships among DEGs was set as high confidence > 0.7.

The promoter analysis performed using Softberry revealed that ParDE and PA1030/PA1029 form a “canonical” genetic organization, wherein the antitoxin gene is tightly adjacent to the downstream toxin gene for co-expression (Salamov and Solovyevand, 2011). Alternatively, the remaining three TA pairs adapt a “reverse” genetic arrangement with the antitoxin gene located at the downstream of the toxin gene (**Figure 1A**). This atypical assembly is usually combined with an additional promoter between the toxin and antitoxin (Fraikin et al., 2020). As expected, the operons of PA1878/PA1879, PA3270/PA3269, and HigBA all remain as second promoters (**Figure 1A**). This mechanism allows a more flexible regulation for overexpression of antitoxins to inhibit toxin expression under a normal condition.

Additionally, we used the STRING database (version 11.5) to excavate the protein–protein interaction (PPI) of type II TAs from *P. aeruginosa* PAO1. The constructed networks reflect the potential pathways associated with these modules (**Figure 1B**) (Szklarczyk et al., 2021). When the parameter of interactive relationships among DEGs was set as high confidence > 0.7, the final PPI network consisted of 51 genes and 121 interlinks. Both PA3270 and PA1878 were interlinked with the *arg* operon, a cluster for arginine biosynthesis, and the PA0124 and PA1030 networks were linked through PA1765. Notably, the HigBA pair was separate from other TA systems and was mostly interlinked with proteases such as Lon and Clp, consistent with the previous study, which exhibited that the HigBA system is associated with Lon protease (Li et al., 2016). Moreover, ParE was involved in lipid biosynthesis and transport processes, and PA1030 was associated with flagellar biosynthesis and Arc1-3 clusters in defense against different threats. As a HD domain-containing protein that belongs to metal-dependent phosphohydrolase, PA1878 participates in alginate synthesis and oleate-diol synthase. In addition to hexosamine metabolism and arginine biosynthesis, PA3270 is also interlinked to the iron uptake transporter FecA, which is consistent with our previous study finding that PA3270 plays a vital role in iron uptake regulation (Song et al., 2022). Therefore, the network analysis revealed multiple functional roles of five TA systems in *P. aeruginosa*.

### Structure modelling and comparative structural analyses describes the various features and evolutionary conservation of the selected type II toxins

Protein structure prediction is promising in understanding the molecular mechanism and biological activity of proteins. Currently, only ParE and PA3270 structures have been explained, and the understanding about the structures of other toxins is limited. To examine the common structural features and key residues of type II TA toxins, the AlphaFold database was used for quaternary structure modeling and deducing protein activity (Jumper et al., 2021). Based on the high sequence identity (31%–46%) with structures of available proteins including 4DW1, 4L1J, and 8F8S, respectively, the resultant models had a per-residue confidence score (pLDDT) of >90, which indicated very high-quality models.

**Figure 2:**
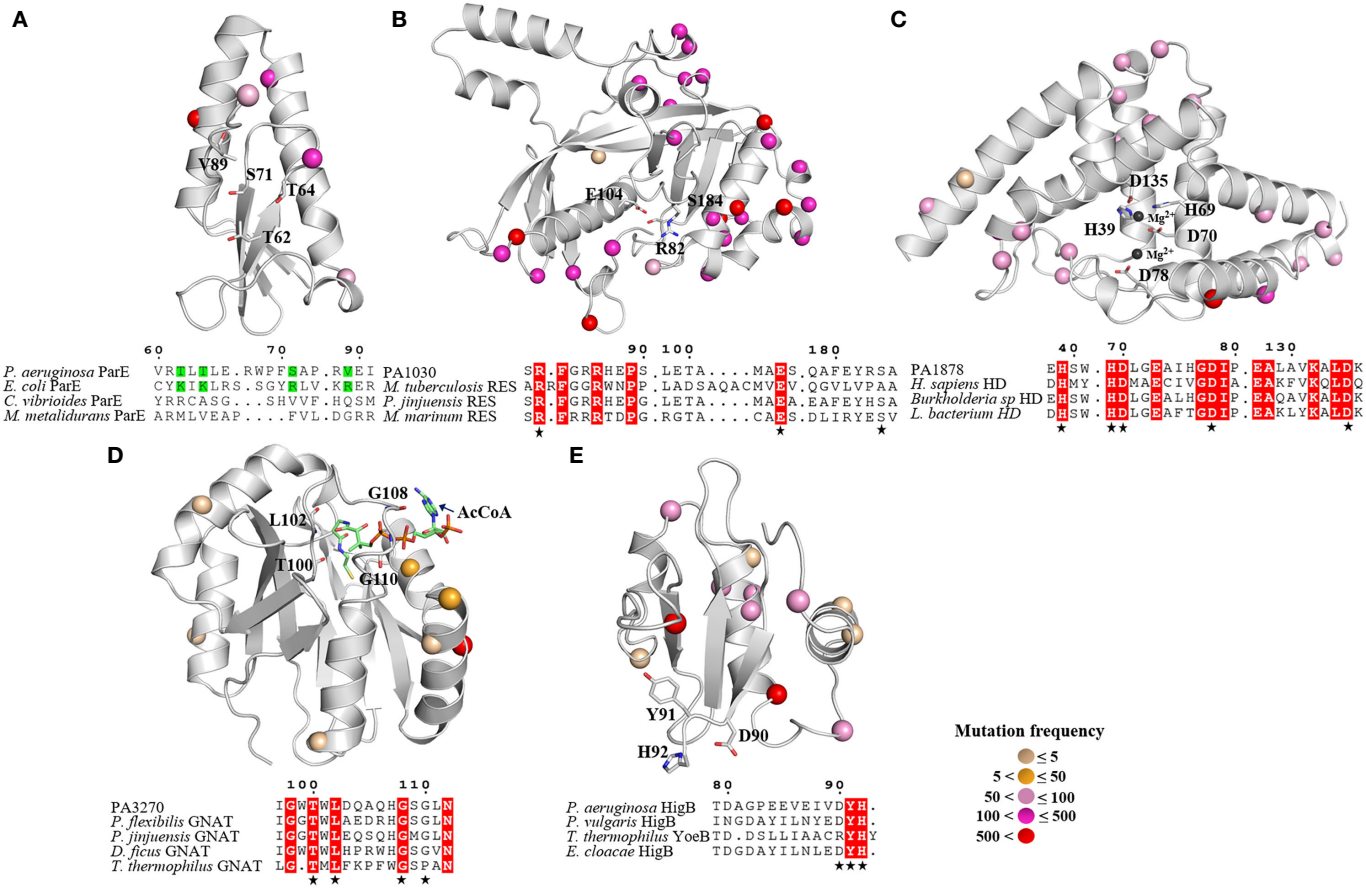
Overall structures of five toxins and their conserved catalysis motifs. **(A)** ParE, **(B)** PA1030, **(C)** PA1878, **(D)** PA 3270 and **(E)** HigB. The 3D structure models of PA1030, PA1878 and HigB are obtained from AlphaFold, whereas the structures of ParE (6XRW) and PA3270 (1YRE) are obtained from RCSB PDB. The mutant sites of toxins from isolates are labeled in sphere style, and the different colors represent mutation frequency. **(A)** Sequence alignment of the *P. aeruginosa* ParE, *E coli* ParE, *Caulobacter vibrioides* ParE and *Mesorhizobium metalidurans* ParE **(B)** Sequence alignment of the PA1030, *M. tuberculosis* Rv1989c, *Pseudomonas jinjuensis* A0A1H0AAW9, *Methylocaldum marinum* A0A250KUB3. **(C)** Sequence alignment of the PA1878, Homo sapiens HDDC2, *Burkholderia sp* A0A3N8LA50, and *Lachnospiraceae bacterium* A0A1H3YLD1. **(D)** Sequence alignment of PA3270, *Pseudomonas jinjuensis* A0A1H0A919, *Pseudomonas flexibilis* A0A0B3C4T8, *Deinococcus ficus* A0A221ST89, *Thermus thermophilus* Q5SHD1. **(E)** Sequence alignment of *P. aeruginosa* HigB, *P. vulgaris* HigB, *Thermus thermophilus* YoeB, *Enterobacter cloacae* HigB. The catalytic residues are indicated as sticks in secondary structure and highlighted (asterisk) in sequence alignment.

Although ParE has a conserved secondary structure consistent with that of the RelE superfamily, it functions to inhibit gyrase-mediated DNA supercoiling rather than cleave mRNA (Muthuramalingam et al., 2019; Snead et al., 2021). Regarding its structure, ParE consists of two β-sheets and four α-helices, this helix-turn-helix motif is found in RelE-type toxins and comes in contact with ribosomal RNA **Figure 2A**). Although ParE exhibits a remarkable conservation of structure through superposition with the structures of the other known ParE family members, the sequence similarity is <31% and the catalytic residues in *Escherichia coli* ParE2 are absent in *P. aeruginosa* ParE. A comparison with the closest structural homolog from *E. coli* revealed that the possible active sites of *P. aeruginosa* ParE are T62, T64, S71, and V89.

Similar to the *Mycobacterium tuberculosis* toxin MbcT, PA1030 is a new RES-type toxin that triggers persister formation by catalyzing NAD^+^ degradation (Zhou et al., 2021). PA1030 has a sandwich fold consisting of nine β strands connected by five α helices, while the central substrate-binding pocket is formed by three conserved residues R82, E104, and S184 (**Figure 2B**).

The PA1878 toxin has a HD domain that defines a superfamily of metal-dependent phosphohydrolases with a doublet of divalent-cation-coordinating His and Asp residues (Huynh et al., 2015). PA1878 folds into an all-helical structure that consists of seven α helices. In PA1878, two central parallel helices interact with other helices located on both sides, and the HD motif is located in the core segment. The arrangement of HD domain helices is such that a large depression is formed on one face of the structure, where the potential metal ions and substrates are bound. Based on the structure of the human HD protein, we noted that the conserved residues including H39, H69, D70, D78, and D135 are potential catalytic sites for interaction with two metal ions.

PA3270 encodes an acetyltransferase belonging to the GCN5-related N-acetyltransferase (GNAT) family and exhibits a conserved GNAT fold with a unique motif (T-X-L-X_5_-G-X-G) for acetyl coenzyme A binding (**Figure 2D**) (Song et al., 2022). Overall, PA3270 consists central, mixed β sheets besieged by two or three α helices on each side. This structure resembles that of its homologous AtaT from *E. coli*, TacT from *Salmonella*, and KacT from *K. pneumoniae*.

The ribosome-dependent endonuclease HigB has a similar fold as the RelE family that cleaves ribosome-bound mRNAs in response to stress (**Figure 2E**). Overall, HigB is formed by three β sheets and three α helices, whereas its C-terminal residues D90, Y91, and H92 are essential for recognizing the ribosomal A site based on the complex of the ribosome-bound *Proteus vulgaris* HigB (Mansour et al., 2022).

Intriguingly, the mutation analysis revealed that most mutant sites were located on the toxin’s surface far away from the active centers, whereas the key residues in active pockets were extremely conserved among the isolates (**Figure 2**). Consequently, the essential roles of bacterial toxins are retained, leading to tolerance to more mutations without affecting the native functions. Moreover, the high mutation rate on the toxin’s surface suggests that the TA interaction as well as the toxin targets may be distinct in *P. aeruginosa* isolates.

**Figure 3:**
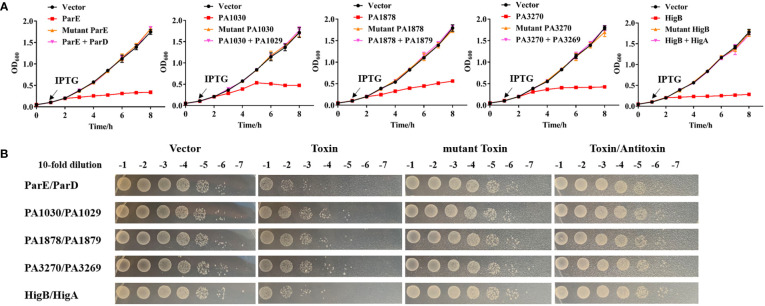
Toxicity assays of toxins and their cognate antitoxins. **(A)** Expression of the toxin causes notable growth arrest of *E coli*, and the cognate antitoxin can neutralize the toxic effects. Overnight cultures of *E coli* BL21 (DE3) harboring pRSFDuet-1 -based plasmids with toxin, antitoxin or mutant toxin genes were reinoculated into fresh LB at OD_600_ = 0.05. After 1 h, the cultures were added by 0.1 mM IPTG and continued to cultivate at 37°C for growth detection. **(B)** After 8 h growth in **(A)**, the cultures were also serially diluted and spotted on LB plates at 37°C for 16 h before colony counting. The detailed mutations about toxins are ParE (T62/A, T64/A, S71/A, V89/A), PA1030 (R82/A, E104/A, S184/A), PA1878 (H39/A, H69/A, D70/A), PA3270 (G108/A, T100/A), HigB (H92/A) referring to the key resides of toxins in **Figure 2**. The experiments were repeated three times.

To further assess the toxicity of the five potential TA pairs as well as their key residues, we repeated neutralization assays in *E. coli* strains. In these strains, the toxin and antitoxin genes were cloned into the pRSFDuet-1 plasmid to construct overexpression systems. Cells expressing different toxins led to a notable decrease in cell growth, whereas cell growth was not affected by the expression of mutated toxins or co-expression of cognate antitoxins (**Figures 3A, B**). In addition, ParE and HigB belonging to the RelE/ParE family led to faster growth inhibition than other toxins (**Figure 3A**), possibly because of the direct interference at the post-transcriptional level. Collectively, according to these results, the five TA systems are widely distributed among *P. aeruginosa* clinical isolates and the conserved active residues of toxins are crucial for full activities.

### Toxins ParE, PA1030 and HigB are important for the antibiotic-induced persistence

Recent evidence has demonstrated that some type II TA systems would be activated with antibiotic treatments, and the expression levels of toxins are related to persister cell formation and therefore their evaluation would be beneficial (Li et al., 2016; Kamruzzaman et al., 2021; Song et al., 2021a). For instance, *higBA* operon expression was induced to trigger persister formation with ciprofloxacin treatment (Li et al., 2016). We next asked determined whether the five TA systems contributed to antibiotic resistance. Strains with toxin gene deletion were constructed, and MBC-B assays were conducted to detect resistance to antibiotics commonly used in clinical therapy against *P. aeruginosa* infections, including meropenem, tobramycin, azithromycin, gentamicin, ciprofloxacin, polymyxin B, and cephalosporin. The results showed no significant difference between the mutants and WT strains in all antibiotic treatments (**Table S4**), indicating no direct contribution of these TA pairs to antibiotic resistance.

**Figure 4:**
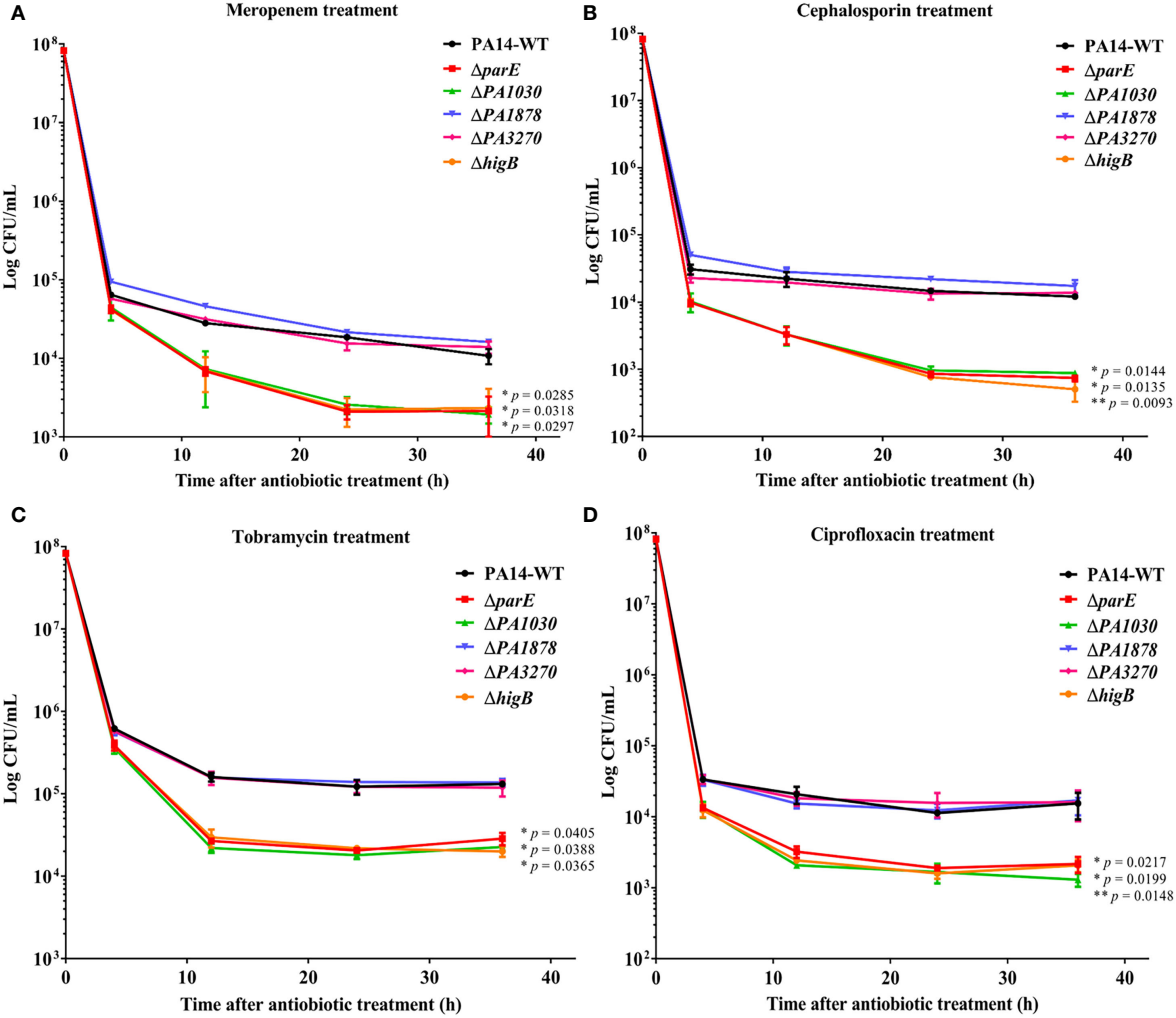
The ParE, PA1030, and HigB toxins increase the levels of antibiotic-tolerant persisters in *P. aeruginosa*. Overnight bacterial cultures of WT *P. aeruginosa* and mutants were re-inoculated into fresh LB medium at OD_600_ = 0.1 and grown to an OD_600_ of 1.0. Then the bacterial cultures were exposed to 50 µg/ml meropenem **(A)**, 100 µg/ml cephalosporin **(B)**, 5 µg/mL tobramycin **(C)** and 5 µg/mL ciprofloxacin **(D)**. Cell viability (CFU/ml) was determined by serial dilution and plating at the time points. The plate was incubated at 37°C for 16 h before colony counting. The results are shown as time-kill curves and expressed as the means ± s.d. of 3 independent experiments (n = 3). Statistical significance was calculated with a two-tailed unpaired t-test. *p < 0.05; **p < 0.01 compared to survival of the WT strain at 36 h.

Persister cells are formed at high lethal concentrations of antibiotics, and type II TA systems are primarily responsible for inducing a dormancy state to enable cells to escape the effects of antibiotics (Mulcahy et al., 2010; Wang and Wood, 2011). Therefore, we examined the role of toxins in *P. aeruginosa* tolerance and persistence by challenging the WT and mutant strains with 50 µg/ml meropenem, 100 µg/ml cephalosporin, 5 µg/ml tobramycin or 5 µg/ml ciprofloxacin (**Figure 4**). As evidenced by the biphasic killing curve with a subpopulation of surviving persisters, *parE*, *PA1030*, and *higB* deletion reduced the bacterial survival rate by approximately 80-fold after meropenem treatment compared to the WT strains, whereas *PA1878* and *PA3270* deletion had no effect on bacterial survival rates under these antibiotic treatments (**Figure 4A**). In addition, similar results were observed under cephalosporin and other antibiotic treatments (**Figures 4B–C, D**). These results thus suggest the crucial roles of *parE*, *PA1030*, and *higB* in persister formation against antibiotics.

### Gene expression profiling of the type II TA modules in *Pseudomonas aeruginosa* cultured in SCFM or during cellular infection

**Figure 5:**
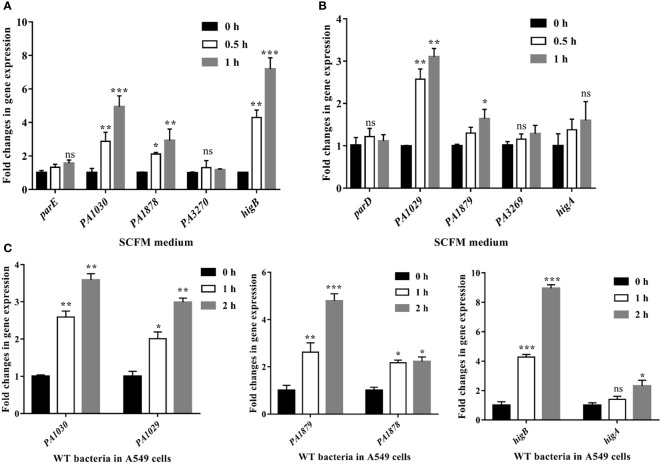
The *PA1030*, *PA1878* and *higB* are activated in SCFM medium and cell adhesion process. Relative mRNA levels of toxins **(A)** and antitoxins **(B)** in SCFM. Overnight bacterial cultures of WT *P. aeruginosa* grown in LB medium were re-inoculated into fresh SCFM at OD_600_ = 0.5 for next culture at 37°C for 0.5 or 1 h. Then the bacteria cultures were collected and the bacterial RNA was extracted for qRT-PCR assays. **(C)** Relative gene expression levels of *PA1030*, *PA1878*, *higB* and their cognate antitoxins in bacterial infection upon A549 cells. The A549 cells were seeded in a 6-well tissue culture plate and then were infected with *P. aeruginosa* PA14 at an MOI of 100 for 1 and 2 h, respectively. The A549 cells were treated with 1% Triton X-100 for 15 min, washed with PBS twice, and then treated with DNase for another 15 min. The bacteria were collected for RNA isolation and qRT-PCR. *p < 0.05; **p < 0.01; ***p < 0.001 by Student’s *t*-test. ns indicates no significant difference.

Studies involving both *in vitro* and *in vivo* approaches are of the opinion that the type II TA system is required for stress responses and benefits pathogenesis within bacteria (Fraikin et al., 2020; Równicki et al., 2020; Song et al., 2022). VapBC from *M. tuberculosis* and Hma-TomB from *S. Typhimurium* could modulate the host immune response to evade killing by the immune system (Agarwal et al., 2020). Being an opportunistic pathogen, *P. aeruginosa* must colonize the sputum (mucus) layer of the CF lung to obtain carbon and energy for future proliferation during chronic infection (Davies, 2002). To address the roles played by TA systems during chronic colonization, we conducted qRT-PCR experiments to measure the gene expression levels of the five TA operons in the synthetic CF sputum medium (SCFM) that mimics the nutritional composition of the CF sputum (Palmer et al., 2007). The gene expression of *PA1030*, *PA1878*, and *higB* was 2.8-, 2.2-, and 4.1-fold upregulated at 0.5 h and 4.9-, 3.1-, and 7.4-fold upregulated at 1 h in the SCFM relative to the initial stage, respectively (**Figure 5A**). The gene expression of the cognate toxin was also significantly upregulated in the SCFM (**Figure 5B**). Additionally, the gene expression of *parE*, *PA3270*, and their antitoxins exhibited no obvious upregulation or downregulation.

To further quantify whether these TA systems would be activated during invasion, we isolated total bacterial RNA from the human alveolar type II epithelial cells (A549) after *P. aeruginosa* infection for 1 or 2 h and then subjected it to qRT-PCR. Compared with those in the sterile PBS buffer-treated bacteria, the expression levels of *PA1030*, *PA1878*, and *higB* were increased by 2.6-, 2.4-, and 3.45-fold at 1 h post-infection, respectively (**Figure 5C**). At 2 h post-infection, *PA1030*, *PA1878*, and *higB* exhibited 2.97-, 4.8-, and 8.85-fold increases, respectively (**Figure 5C**). Expression levels of the cognate toxin genes including *PA1029* and *PA1879* were also upregulated. Therefore, our results indicated that PA1030/PA1029, PA1878/PA1879, and HigBA systems are activated both in the SCFM medium and during infection.

### Toxins PA1030 and HigB contribute to the bacterial intracellular survival in macrophages

**Figure 6:**
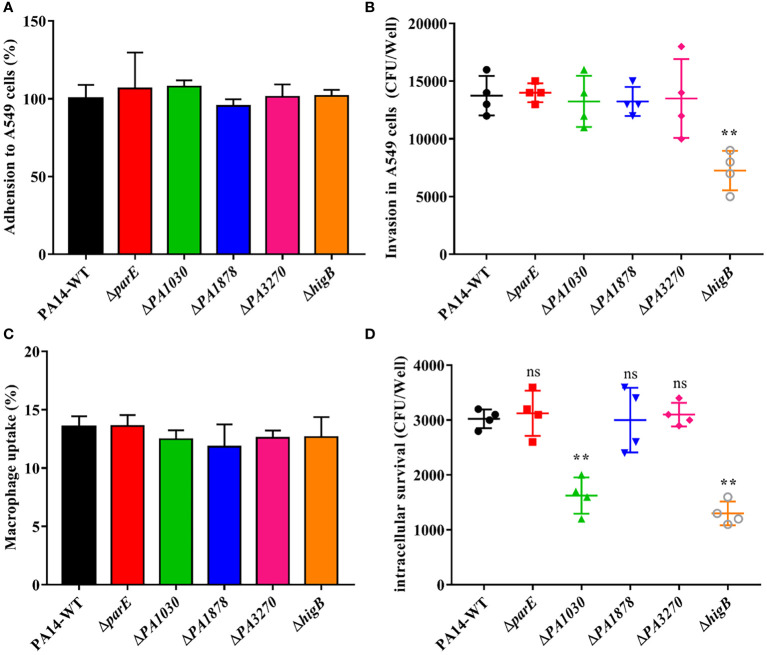
Adhesion and invasion in epithelial cell A549 and Macrophage uptake and survival assay of WT *P. aeruginosa* and mutants. **(A)** Adhesion and **(B)** Invasion assay of WT and mutants in human alveolar type II epithelial cell A549. **(C)** Uptake and **(D)** % Survival of WT and mutants in RAW264.7 macrophages. Sterile PBS served as negative control. All experiments were performed in triplicates and error bars represent error of the mean (±) for SD. *p < 0.05; **p < 0.01; ***p < 0.001 by One-way ANOVA statistical test. ns indicates no significant difference.

The activated toxin of the TA system in pathogens such as *S. typhimurium* and *M. tuberculosis* may aid the spread of non-replicating persister cells and intracellular survival within the host intracellular milieu (Lobato-Márquez et al., 2015; Agarwal et al., 2020). Because *PA1030*, *PA1878*, and *higB* were activated during invasion, we studied the roles of TA systems in invasion and intracellular survival. For this purpose, the A549 cells were infected with *P. aeruginosa* WT and mutant strains and lysed for bacterial cell counting. The mutants showed no significant difference in their adhesion ability to epithelial cells, whereas the Δ*higB* strain (52.7%) exhibited significantly reduced invasion compared with the WT strain (**Figures 6A, B**). Studies have proven that *higB* is required for upregulating T3SS-associated effectors. This may explain the attenuation of the invasion ability with *higB* deletion.

Given that macrophages are among the first lines of defense against microbial invasion in the lower airways, the mutant and WT strains were also assessed in uptake and survival assays within RAW264.7 murine macrophage cells (Kooguchi et al., 1998). Toxin gene deletion had no effect on uptake by macrophages compared with WT at 2 h post-infection. However, intracellular survival was significantly reduced in the Δ*higB* (63.2%) and Δ*PA1030* (57.8%) strains at 24 h post-infection (**Figures 6C, D**), indicating the positive roles of HigB and PA1030 in macrophage defense. Overall, these results confirmed the pivotal role played by PA1030/PA1029 and HigBA TA systems in *P. aeruginosa* virulence and emphasized that PA1030 and HigB can act as potential targets for infection treatments.

## Discussion

*P. aeruginosa* is an emerging opportunistic pathogen and is ranked fourth in total clinical isolates according to the 2021 data from the China Antimicrobial Surveillance Network (CHINET) (Feng et al., 2021; Tenover et al., 2022). The survival of *P. aeruginosa* persisters in the presence of antibiotics is believed to be among the primary reasons for most clinical isolates to exhibit high resistance to antibiotics (Mulcahy et al., 2010). The type II TA system has been reported to be a vital player in persister cell formation in other pathogenic bacteria, but little is known about persister cell formation in *P. aeruginosa* (Wang and Wood, 2011; Fasani and Savageau, 2013). For a better understanding about the mechanisms underlying persistence in *P. aeruginosa* isolates, we analyzed the diversity of five genomic type II TA systems among these isolates and their distinct structural features. All toxins and antitoxins were widely distributed among 4955 clinical isolates of *P. aeruginosa* and share a high sequence identity, except *higB*, indicating the unusual roles of HigA in *P. aeruginosa*. Intriguingly, the mutation analysis of toxins revealed that most mutant sites were located on the toxin surface far away from the active centers and the key residues were extremely conserved among the isolates. This indicated the conserved functional mechanisms of these toxins. We also screened the *P. aeruginosa* mutants to identify the toxin that contributes to the tolerance against diverse antibiotics. The results showed that deletion of type II toxins had no effect on direct antibiotic resistance in MBC-B assays, whereas *parE*, *PA1030*, and *higB* contributed to persister formation after lethal antibiotic treatments. Further, gene transcription analysis revealed that PA1030/PA1029, PA1878/PA1879, and HigBA systems were specifically activated both in the SCFM medium and during invasion. In addition, PA1030 and HigB were essential for intracellular survival in macrophages. Thus, additional studies are urgently required to explore the detailed functions of PA1030/PA1029 and HigBA systems in persister formation and virulence.

Studies have mostly focused on the functional roles of type II TA systems in standard strains of well-known pathogens, while the diversity and prevalence of these systems among clinical isolates remain to be completely characterized. In *P. aeruginosa* standard strains and other clinical isolates, the five type II TA systems were widely distributed in genomes and highly conservative in central key residues but variable in surface residues. Our study confirms the major contributions of key residues to full activities of toxins, thereby suggesting the high tolerance of the TA system as a counter to frequent mutations during *P. aeruginosa* transmission. It also reveals the TA interaction and that the targets of toxins may be distinct in *P. aeruginosa* isolates. In addition, *higB* rather than *higA* was lost in some of the isolated strains. The toxin gene was also absent in *Acetobacter* species, indicating more potential functions of HigA.

A growing body of evidence indicates that dormancy and persistence are associated with growth arrest caused by increasing expression levels of toxins. Although all toxins from the five TA systems exhibited notable toxic effects on *E. coli* cells, easily overlooked difference was found in this experiment. In this case, ParE and HigB, which directly halt DNA replication or protein translation, exhibited a stronger growth inhibitory ability. The persistence against antibiotics was also attenuated in strains with *parE* and *higB* deletion. On the contrary, contributions of other toxins, such as PA1878 and PA3270 to persiter cell formation were limited.

Despite the initial functions related to the maintenance of plasmids and persistence, type II TA systems exert various biological functions, including abortive infection, biofilm formation and gene regulation (Fraikin et al., 2020; Kamruzzaman et al., 2021; Xia et al., 2021). Based on the PPI network generated using the STRING database, numerous cross-interlinks were discovered between four TA systems, whereas the HigBA system was different from others. Evidences have highlighted the virulence contribution of HigBA system in *P. aeruginosa* (Li et al., 2016; Wood and Wood, 2016), and recent studies further confirmed that HigA could directly regulate the expression of virulence-associated genes (Guo et al., 2019; Song et al., 2021b). PA1030 was associated with flagellar biosynthesis and Arc1-3 clusters in defense against external threats, and Zhou et al. also demonstrated that this TA system modulate the persister formation by reducing the intracellular NAD^+^ level in *P. aeruginosa* (Zhou et al., 2021). While PA1878 and PA3270 were involved in amino acid metabolism. *parE* and *PA3270* were not activated during bacterial invasion, but studies have demonstrated that *parE* protects cells challenged with anti-gyrase antibiotics. Moreover, pa3270 was activated to regulate the expression of iron uptake genes under iron starvation (Muthuramalingam et al., 2019; Song et al., 2022). These data suggest that these TA systems have different roles in more biological pathways.

TA systems are more abundant in pathogens associated with severe or chronic infections than in nonpathogenic bacteria (Fraikin et al., 2020; Gu et al., 2021; Kamruzzaman et al., 2021). The VapBC22 TA pair in *M. tuberculosis* is associated with a key regulatory network for modulating bacterial pathogenesis and the host immune response (Agarwal et al., 2020). Loss of the VapC22 toxin reduces the expression of virulence-associated proteins, making them more susceptible to oxidative stress and attenuating growth in host tissues. MazEF is believed to regulate the high antibiotic tolerance phenotype of *S. aureus*, a major gram-positive organism that causes surgical implant infections (Ma et al., 2019). *mazF* deletion in *S. aureus* elevated biofilm growth and virulence but decreased antibiotic tolerance. Consistently, we have proven that PA1030/PA1029 and HigBA are responsible for persistence and virulence in *P. aeruginosa*, which are potential targets for novel drug development. However, the current data about the regulatory mechanism and structures are unclear and insufficient. Moreover, the specific functions of other type II TA systems need to be explored. Thus, additional studies are required to unveil how external stresses trigger these systems and intracellular targets of toxins, and what are the driving forces for the numerous TA systems that exist in pathogens.

## Data availability statement

The datasets presented in this study can be found in online repositories. The names of the repository/repositories and accession number(s) can be found in the article/**Supplementary Material**.

## Author contributions

YS conceived and designed the experiments. YS and HT carried out qRT-PCR and invasion assays. YS and RB performed the structure modeling and data analysis. YS, HT and RB wrote the manuscript. HT and RB provided supervision. All authors contributed to the article and approved the submitted version.

## References

[B1] Agarwal S. Sharma A. Bouzeyen R. Deep A. Sharma H. Mangalaparthi K. K.. (2020). VapBC22 toxin-antitoxin system from mycobacterium tuberculosis is required for pathogenesis and modulation of host immune response. Sci. Adv. 6 (23), eaba6944. doi: 10.1126/sciadv.aba6944 32537511 PMC7269643

[B2] Alonso J. C. (2021). Toxin–antitoxin systems in pathogenic bacteria. Toxins 13 (2), 74. doi: 10.3390/toxins13020074 33498357 PMC7909440

[B3] Davies J. C. (2002). Pseudomonas aeruginosa in cystic fibrosis: Pathogenesis and persistence. Paediatric Respir. Rev. 3 (2), 128–134. doi: 10.1016/S1526-0550(02)00003-3 12297059

[B4] Fasani R. A. Savageau M. A. (2013). Molecular mechanisms of multiple toxin–antitoxin systems are coordinated to govern the persister phenotype. Proc. Natl. Acad. Sci. 110 (27), E2528–E2537. doi: 10.1073/pnas.1301023110 23781105 PMC3703989

[B5] Feng W. Huang Q. Wang Y. Yuan Q. Li X. Xia P.. (2021). Changes in the resistance and epidemiological characteristics of pseudomonas aeruginosa during a ten-year period. J. Microbiol. Immunol. Infection 54 (2), 261–266. doi: 10.1016/j.jmii.2019.08.017 31628088

[B6] Fraikin N. Goormaghtigh F. Van Melderen L. (2020). Type II toxin-antitoxin systems: Evolution and revolutions. J. bacteriol. 202 (7), e00763–e00719. doi: 10.1128/JB.00763-19 31932311 PMC7167474

[B7] Golmoradi Zadeh R. Mirshekar M. Sadeghi Kalani B. Pourghader J. Barati M. Masjedian Jazi F. (2022). The expression of type II TA system genes following persister cell formation in pseudomonas aeruginosa isolates in the exponential and stationary phases. Arch. Microbiol. 204 (8), 1–10. doi: 10.1007/s00203-022-03038-x 35781545

[B8] Gu Q. He P. Wang D. Ma J. Zhong X. Zhu Y.. (2021). An auto-regulating type II toxin-antitoxin system modulates drug resistance and virulence in streptococcus suis. Front. Microbiol. 2333. doi: 10.3389/fmicb.2021.671706 PMC8406773 34475853

[B9] Guo Y. Sun C. Li Y. Tang K. Ni S. Wang X. (2019). Antitoxin HigA inhibits virulence gene mvfR expression in pseudomonas aeruginosa. Environ. Microbiol. 21 (8), 2707–2723. doi: 10.1111/1462-2920.14595 30882983

[B10] Helaine S. Cheverton A. M. Watson K. G. Faure L. M. Matthews S. A. Holden D. W. (2014). Internalization of salmonella by macrophages induces formation of nonreplicating persisters. Science 343 (6167), 204–208. doi: 10.1126/science.1244705 24408438 PMC6485627

[B11] Huynh T. N. Luo S. Pensinger D. Sauer J.-D. Tong L. Woodward J. J. (2015). An HD-domain phosphodiesterase mediates cooperative hydrolysis of c-di-AMP to affect bacterial growth and virulence. Proc. Natl. Acad. Sci. 112 (7), E747–E756. doi: 10.1073/pnas.1416485112 25583510 PMC4343097

[B12] Jumper J. Evans R. Pritzel A. Green T. Figurnov M. Ronneberger O.. (2021). Highly accurate protein structure prediction with AlphaFold. Nature 596 (7873), 583–589. doi: 10.1038/s41586-021-03819-2 34265844 PMC8371605

[B13] Jurėnas D. Fraikin N. Goormaghtigh F. Van Melderen L. (2022). Biology and evolution of bacterial toxin–antitoxin systems. Nat. Rev. Microbiol. 20 (6), 335–350. doi: 10.1038/s41579-021-00661-1 34975154

[B14] Kamruzzaman M. Wu A. Y. Iredell J. R. (2021). Biological functions of type II toxin-antitoxin systems in bacteria. Microorganisms 9 (6), 1276. doi: 10.3390/microorganisms9061276 34208120 PMC8230891

[B15] Kooguchi K. Hashimoto S. Kobayashi A. Kitamura Y. Kudoh I. Wiener-Kronish J.. (1998). Role of alveolar macrophages in initiation and regulation of inflammation in pseudomonas aeruginosa pneumonia. Infection Immun. 66 (7), 3164–3169. doi: 10.1128/IAI.66.7.3164-3169.1998 PMC108328 9632581

[B16] Li M. Long Y. Liu Y. Liu Y. Chen R. Shi J.. (2016). HigB of pseudomonas aeruginosa enhances killing of phagocytes by up-regulating the type III secretion system in ciprofloxacin induced persister cells. Front. Cell. infect. Microbiol. 6, 125. doi: 10.3389/fcimb.2016.00125 27790409 PMC5064212

[B17] Lobato-Márquez D. Moreno-Córdoba I. Figueroa V. Díaz-Orejas R. García-del Portillo F. (2015). Distinct type I and type II toxin-antitoxin modules control salmonella lifestyle inside eukaryotic cells. Sci. Rep. 5 (1), 1–10. doi: 10.1038/srep09374 PMC4366850 25792384

[B18] Lyczak J. B. Cannon C. L. Pier G. B. (2000). Establishment of pseudomonas aeruginosa infection: Lessons from a versatile opportunist. Microbes infect. 2 (9), 1051–1060. doi: 10.1016/S1286-4579(00)01259-4 10967285

[B19] Ma D. Mandell J. B. Donegan N. P. Cheung A. L. Ma W. Rothenberger S.. (2019). The toxin-antitoxin MazEF drives staphylococcus aureus biofilm formation, antibiotic tolerance, and chronic infection. MBio 10 (6), e01658–e01619. doi: 10.1128/mBio.01658-19 31772059 PMC6879715

[B20] Mah T.-F. (2014). Establishing the minimal bactericidal concentration of an antimicrobial agent for planktonic cells (MBC-p) and biofilm cells (MBC-b). JoVE (Journal Visualized Experiments) 83), e50854. doi: 10.3791/50854 PMC4047662 24430536

[B21] Mansour M. Giudice E. Xu X. Akarsu H. Bordes P. Guillet V.. (2022). Substrate recognition and cryo-EM structure of the ribosome-bound TAC toxin of mycobacterium tuberculosis. Nat. Commun. 13 (1), 1–14. doi: 10.1038/s41467-022-30373-w 35552387 PMC9098466

[B22] Mulcahy L. R. Burns J. L. Lory S. Lewis K. (2010). Emergence of pseudomonas aeruginosa strains producing high levels of persister cells in patients with cystic fibrosis. J. bacteriol. 192 (23), 6191–6199. doi: 10.1128/JB.01651-09 20935098 PMC2981199

[B23] Muthuramalingam M. White J. C. Murphy T. Ames J. R. Bourne C. R. (2019). The toxin from a ParDE toxin-antitoxin system found in pseudomonas aeruginosa offers protection to cells challenged with anti-gyrase antibiotics. Mol. Microbiol. 111 (2), 441–454. doi: 10.1111/mmi.14165 30427086 PMC6368863

[B24] Palmer K. L. Aye L. M. Whiteley M. (2007). Nutritional cues control pseudomonas aeruginosa multicellular behavior in cystic fibrosis sputum. J. bacteriol. 189 (22), 8079–8087. doi: 10.1128/JB.01138-07 17873029 PMC2168676

[B25] Paul P. Patel P. Verma S. K. Mishra P. Sahu B. R. Panda P. K.. (2022). The hha–TomB toxin–antitoxin module in salmonella enterica serovar typhimurium limits its intracellular survival profile and regulates host immune response. Cell Biol. Toxicol. 38 (1), 111–127. doi: 10.1007/s10565-021-09587-z 33651227

[B26] Pinel-Marie M.-L. Brielle R. Riffaud C. Germain-Amiot N. Polacek N. Felden B. (2021). RNA Antitoxin SprF1 binds ribosomes to attenuate translation and promote persister cell formation in staphylococcus aureus. Nat. Microbiol. 6 (2), 209–220. doi: 10.1038/s41564-020-00819-2 33398097

[B27] Qin S. Xiao W. Zhou C. Pu Q. Deng X. Lan L.. (2022). Pseudomonas aeruginosa: Pathogenesis, virulence factors, antibiotic resistance, interaction with host, technology advances and emerging therapeutics. Signal Transduction Targeted Ther. 7 (1), 1–27. doi: 10.1038/s41392-022-01056-1 PMC9233671 35752612

[B28] Rio D. C. Ares M. Hannon G. J. Nilsen T. W. (2010). Purification of RNA using TRIzol (TRI reagent). Cold Spring Harbor Protoc. 2010 (6), pdb. prot5439. doi: 10.1101/pdb.prot5439 20516177

[B29] Równicki M. Lasek R. Trylska J. Bartosik D. (2020). Targeting type II toxin–antitoxin systems as antibacterial strategies. Toxins 12 (9), 568. doi: 10.3390/toxins12090568 32899634 PMC7551001

[B30] Salamov V. S. A. Solovyevand A. (2011). Automatic annotation of microbial genomes and metagenomic sequences. Metagenomics its Appl. agriculture biomed. Environ. Stud., 61–78.

[B31] Sato H. Okinaga K. Saito H. (1988). Role of pili in the pathogenesis of pseudomonas aeruginosa burn infection. Microbiol. Immunol. 32 (2), 131–139. doi: 10.1111/j.1348-0421.1988.tb01372.x 2897617

[B32] Snead K. J. Moore L. L. Bourne C. R. (2021). ParD antitoxin hotspot alters a disorder-to-order transition upon binding to its cognate ParE toxin, lessening its interaction affinity and increasing its protease degradation kinetics. Biochemistry 61 (1), 34–45. doi: 10.1021/acs.biochem.1c00584 34914378 PMC9805813

[B33] Song Y. Luo G. Zhu Y. Li T. Li C. He L.. (2021a). Pseudomonas aeruginosa antitoxin HigA functions as a diverse regulatory factor by recognizing specific pseudopalindromic DNA motifs. Environ. Microbiol. 23 (3), 1541–1558. doi: 10.1111/1462-2920.15365 33346387

[B34] Song Y. Zhang S. Luo G. Shen Y. Li C. Zhu Y.. (2021b). Type II antitoxin HigA is a key virulence regulator in pseudomonas aeruginosa. ACS Infect. Dis. 7 (10), 2930–2940. doi: 10.1021/acsinfecdis.1c00401 34554722

[B35] Song Y. Zhang S. Ye Z. Song Y. Chen L. Tong A.. (2022). The novel type II toxin–antitoxin PacTA modulates pseudomonas aeruginosa iron homeostasis by obstructing the DNA-binding activity of fur. Nucleic Acids Res. 50 (18), 10586–10600. doi: 10.1093/nar/gkac867 36200834 PMC9561280

[B36] Szklarczyk D. Gable A. L. Nastou K. C. Lyon D. Kirsch R. Pyysalo S.. (2021). The STRING database in 2021: Customizable protein–protein networks, and functional characterization of user-uploaded gene/measurement sets. Nucleic Acids Res. 49 (D1), D605–D612. doi: 10.1093/nar/gkab835 33237311 PMC7779004

[B37] Tenover F. C. Nicolau D. P. Gill C. M. (2022). Carbapenemase-producing pseudomonas aeruginosa–an emerging challenge. Emerg. Microbes Infect. 11 (1), 811–814. doi: 10.1080/22221751.2022.2048972 35240944 PMC8920394

[B38] Wang X. Wood T. K. (2011). Toxin-antitoxin systems influence biofilm and persister cell formation and the general stress response. Appl. Environ. Microbiol. 77 (16), 5577–5583. doi: 10.1128/AEM.05068-11 21685157 PMC3165247

[B39] Winsor G. L. Griffiths E. J. Lo R. Dhillon B. K. Shay J. A. Brinkman F. S. (2016). Enhanced annotations and features for comparing thousands of pseudomonas genomes in the pseudomonas genome database. Nucleic Acids Res. 44 (D1), D646–D653. doi: 10.1093/nar/gkv1227 26578582 PMC4702867

[B40] Wood T. L. Wood T. K. (2016). The HigB/HigA toxin/antitoxin system of pseudomonas aeruginosa influences the virulence factors pyochelin, pyocyanin, and biofilm formation. Microbiologyopen 5 (3), 499–511. doi: 10.1002/mbo3.346 26987441 PMC4906001

[B41] Wu W. Jin Y. Bai F. Jin S. (2015). “Pseudomonas aeruginosa,” in Molecular medical microbiology (Elsevier), 753–767.

[B42] Xia K. Ma J. Liang X. (2021). Impacts of type II toxin-antitoxin systems on cell physiology and environmental behavior in acetic acid bacteria. Appl. Microbiol. Biotechnol. 105 (11), 4357–4367. doi: 10.1007/s00253-021-11357-0 34021811

[B43] Xie Y. Wei Y. Shen Y. Li X. Zhou H. Tai C.. (2018). TADB 2.0: An updated database of bacterial type II toxin–antitoxin loci. Nucleic Acids Res. 46 (D1), D749–D753. doi: 10.1093/nar/gkx1033 29106666 PMC5753263

[B44] Zhou J. Li S. Li H. Jin Y. Bai F. Cheng Z.. (2021). Identification of a toxin–antitoxin system that contributes to persister formation by reducing NAD in pseudomonas aeruginosa. Microorganisms 9 (4), 753. doi: 10.3390/microorganisms9040753 33918483 PMC8065639

