# Supplementary material for: Comparative analysis of five type II TA systems identified in Pseudomonas aeruginosa reveals their contributions to persistence and intracellular survival
Source: Front Cell Infect Microbiol. 2023 Feb 13;13:1127786. doi: 10.3389/fcimb.2023.1127786 (PMC9948252; doi:10.3389/fcimb.2023.1127786)
Supplement: Supplementary file 1 [file DataSheet_1.pdf]

Supplemental material

Comparative analysis of five type II TA systems identified in *Pseudomonas aeruginosa* reveals their distinct roles in persistence and virulence

Yingjie Song[1], Hong Tang[2*], Rui Bao[2*]

[1]College of Life Science, Sichuan Normal University, Chengdu, China

[2]Division of Infectious Diseases, State Key Laboratory of Biotherapy and Center of Infectious Diseases, West China Hospital, Sichuan University, Chengdu, China

*Correspondence should be addressed to e-mail: Rui Bao: baorui@scu.edu.cn (R.B.); htang6198@hotmail.com;

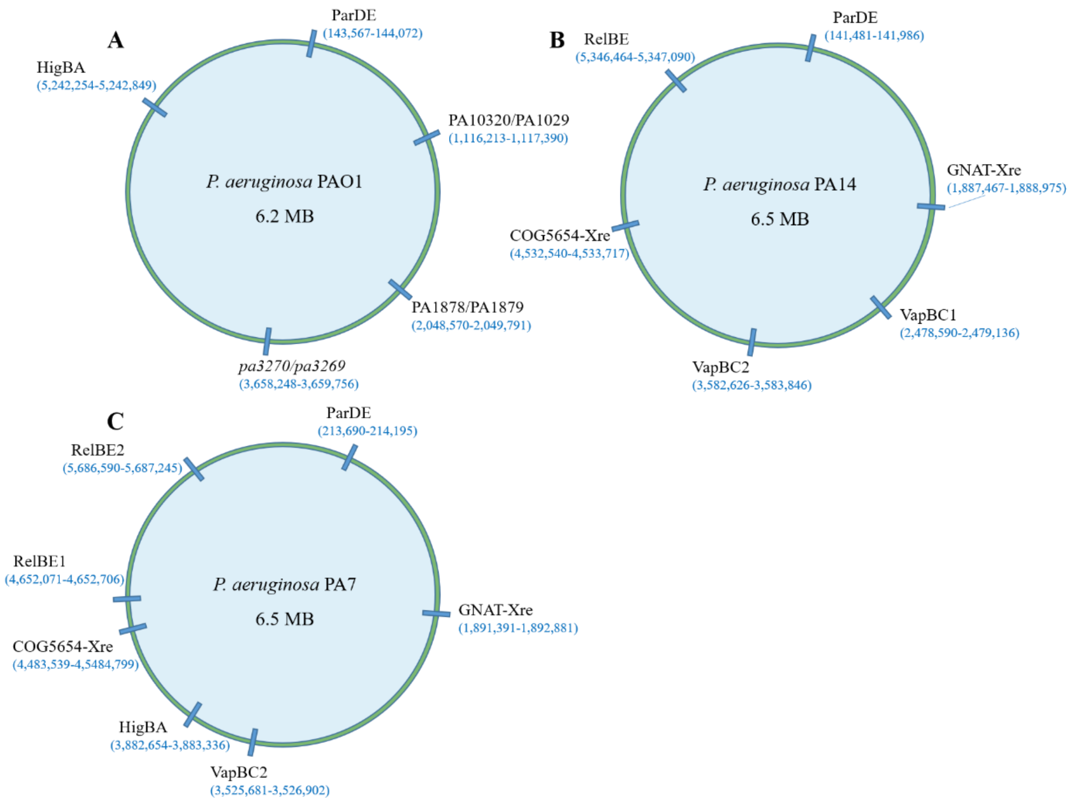

Figure S1. Distribution of type II TA loci *P. aeruginosa*. Bioinformation method based on TADB 2.0 to identify the chromosomal locations of relBE, higBA, parDE, GNAT-Xre, vapBC loci in *P. aeruginosa* PAO1 (A), *P. aeruginosa* PA14 (B), and *P. aeruginosa* PA7 (C).

Table S1. Bacteria strains and plasmids.

Reagent or Resource	Source	Identifier
**Bacterial cells and plasmids**		
E. coli BL21(DE3)	Beijing Genesand Biotech Co.,Ltd	Cat# SEC19
E. coli DH5α	Beijing Genesand Biotech Co.,Ltd	Cat# SCC01
P. aeruginosa PA14	Prof. Wang' Lab	N/A
pME6032	This study	Song et al., 2021
pEX18-Gm	This study	Song et al., 2021
pRSFDuet-1	This study	Song et al., 2021

Table S2. Primers used in the work.

Purpose/Name	Sequence (5'-3')
**Protein Expression**	
**pRSFDuet-*parE*-f**	ACCATCATCACCACAGCCAGGCCTGAAGTGGACCCGCAAGG
**pRSFDuet-*parE*-r**	CTGTTCGACTTAAGCATTATCATCAGGGGGTAATTTCGACTCT
**pRSFDuet-*parD*-f**	AGAAGGAGATATACATATGATGAGCACCGTAGTCTCGTTC
**pRSFDuet-*parD*-r**	GCAGCAGCCTAGGTTAATTACTATCATTGCAGCCCCCAGCGCTTC
**pRSFDuet-*PA1030*-f**	ACCATCATCACCACAGCCAGGTGAGCGAGATCTGGCGACAG
**pRSFDuet-*PA1030*-r**	CTGTTCGACTTAAGCATTATCATCACGCCGGATGCGGCAACT
**pRSFDuet-*PA1029*-f**	AGAAGGAGATATACATATGGTGACGCAGCTCGAACTGGCC
**pRSFDuet-*PA1029*-r**	GCAGCAGCCTAGGTTAATTACTATCAGACCTTGCCGCGGATCGCA
**pRSFDuet-*PA1878*-f**	ACCATCATCACCACAGCCAGATGACCGAGCCTCTCCCTTCTTC
**pRSFDuet-*PA1878*-r**	CTGTTCGACTTAAGCATTATCATCAGAGGGTTTCCACCAGGCGC
**pRSFDuet-*PA1879*-f**	AGAAGGAGATATACATATGATGTCCCTCGAACTGCTGAGC
**pRSFDuet-*PA1879*-r**	GCAGCAGCCTAGGTTAATTACTACTAGGCGTCGCGCCGAACTGC
**pRSFDuet-*PA3270*-f**	ACCATCATCACCACAGCCAGATGTCGAGGAGGGCGCCCATG
**pRSFDuet-*PA3270*-r**	CTGTTCGPPACAAGCATTATCAGCCGGTGAAGCTGGCTTCC
**pRSFDuet-*PA3269*-f**	AGAAGGAGATATACATATGGTGCCGGACGAGACGAGCGG
**pRSFDuet-*PA3269*-r**	GCAGCAGCCTAGGTTAATTACTATTCGTAGATCGTCAAGGATGAGC
**pRSFDuet-*higB*-f**	ACCATCATCACCACAGCCAGATGATTCTGACCTTTCG
**pRSFDuet-*higB*-r**	CTGTTCGACTTAAGCATTATCATCAGTGGTAATCAACTATTTC
**pRSFDuet-*higA*-f**	AGAAGGAGATATACATATGATGGCTACCAATGGTATGCGC
**pRSFDuet-*higA*-r**	GCAGCAGCCTAGGTTAATTACTACTATCCGTGAGCAAGCAGCGG
**Gene knockout**	
**pEX18-*parE*-upstream-f**	AACGACGGCCAGTGCCAAGCTTGAGGTCAAGCCACGCGAAGTG
**pEX18-*parE*-upstream-r**	CTCCTTCTTAAAGTTAAACTGCAGCCCCCAGCGCTTCTC
**pEX18-*parE*-downstream-f**	GTTTAACTTTAAGAAGGAGAAGTGGTCACACGGTTACAAG
**pEX18-*parE*-downstream-r**	TTCGAGCTCGGTACCCGGGGATCCACCGGCGCCGCCAGGCGC
**pEX18-*PA1030*-upstream-f**	AACGACGGCCAGTGCCAAGCTTTGCGGAAGGCTTGCTGGCGGTGAC
**pEX18-*PA1030*-upstream-r**	CTCCTTCTTAAAGTTAAACCTTCCGCCCCTCGCTTCCTGTG
**pEX18-*PA1030*-downstream-f**	GTTTAACTTTAAGAAGGAGGTGGAGGGGGCTCGGTTGCCAGCG
**pEX18-*PA1030*-downstream-r**	TTCGAGCTCGGTACCCGGGGATCAGTTGCGCGGTCGCCAGTCGCTC
**pEX18-*PA1878*-upstream-f**	AACGACGGCCAGTGCCAAGCTTGTCGCTGGCGCCTGCTGCACCG
**pEX18-*PA1878*-upstream-r**	CTCCTTCTTAAAGTTAAACGGCGCGGGCTCCGCTACCGGTGA

**pEX18-*PA1878*-downstream-f**	GTTTAACTTTAAGAAGGAGCGTGCGCCCGCCCGGGCAGCGT
**pEX18-*PA1878*-downstream-r**	TTCGAGCTCGGTACCCGGGGATCCTGGAACCGATCGTCCAGC
**pEX18-*PA3270*-upstream-f**	AACGACGGCCAGTGCCAAGCTTTTGCCGTAGCGGGCGGCGATG
**pEX18-*PA3270*-upstream-r**	CTCCTTCTTAAAGTTAAACCGACGCAAAACTCCGCTC
**pEX18-*PA3270*-downstream-f**	GTTTAPACTTAAGAAGGAGAGCCGGTTCCGTCGCTCCTG
**pEX18-*PA3270*-downstream-r**	TTCGAGCTCGGTACCCGGGGATACGGGCCACCGCCAACGGCTC
**pEX18-*higB*-upstream-f**	AACGACGGCCAGTGCCAAGCTTGTACGACCCTGGGAGCCGATATCC
**pEX18-*higB*-upstream-r**	CTCCTTCTTAAAGTTAAACTCATTAACCCTTAACGTTAAG
**pEX18-*higB*-downstream-f**	GTTTAACTTTAAGAAGGAGATGGCTACCAATGGTATGC
**pEX18-*higB*-downstream-r**	TTCGAGCTCGGTACCCGGGGATGATCAGAAAATTATCTAGC
**qRT-PCR**	
***parE*-f**	GCTCTGAAAGCCGTTCAG
***parE*-r**	CAGGGTCAGGGTGCGTACG
***parD*-f**	GTCTCGTTCCGCGCCGATGA
***parD*-r**	GGTACTGCGCGAGCGCCTG
***PA1030*-f**	GAGCCAAGCCTGTTCTAC
***PA1030*-r**	CAGGACACAACGGTAATACG
***PA1029*-f**	CACTCCCAACCATCAC
***PA1029*-r**	AGGTATTCCAGCACAT
***PA1878*-f**	GCAATCGACCATCTCGCGCTG
***PA1878*-r**	AACAGGCGATGGGTGAGGTCG
***PA1879*-f**	CAACCGCCTCGAATTCCTCC
***PA1879*-r**	GCGCAGGACCTTGCCGAGGTC
***PA3270*-f**	TCAAGCATGCCTTCGACAAC
***PA3270*-r**	AGACGAAGGTGTCGTCTAGC
***PA3269*-f**	TGATCAACCCCGACGAAGTG
***PA3269*-r**	CGCCGCCATGATGGATTTC
***higB*-f**	TCTGACCTTTCGCTGCGACGAG
***higB*-r**	TCCCTGCAACGGCTCCAACC
***higA*-f**	GTATGCGCCCCATCCATCCT
***higA*-r**	CAAAGTAACGCCCCAGACGA

Table S3. Prevalence of five TA loci among *P. aeruginosa* clinical isolates.

TA pair	Size T/A	Prevalence %	Number of strains containing multiple repeat TA loci
ParDE	93/75	100	31
PA1030/PA1029	251/122	100	33
PA1878/PA1879	184/192	99.35	60
PA3270/PA3209	195/284	100%	28
HigBA	92/101	64.90%	8

For each TA pair, its prevalence in % of isolates is stated, and the strains containing multiple copies of the TA pairs are also counted.

Table S4. MBC-B to six antibiotics of the WT *P. aeruginosa* and mutants in developed biofilms.

Antibiotic	MIC (mg/ml)					
	WT	Δ*parE*	Δ*PA1030*	Δ*PA1878*	Δ*PA3270*	Δ*higB*
Meropenem	0.005	0.005	0.005	0.005	0.005	0.005
Tobramycin	0.001	0.001	0.001	0.001	0.001	0.001
Azithromycin	0.2	0.2	0.2	0.2	0.2	0.2
Gentamicin	0.025	0.025	0.025	0.025	0.025	0.025
Ciprofloxacin	0.001	0.001	0.001	0.001	0.001	0.001
Polymyxin B	0.05	0.05	0.05	0.05	0.05	0.05
cephalosporin	0.01	0.01	0.01	0.01	0.01	0.01
